# Probiotic Therapy: A Promising Strategy for the Control of Canine Hookworm

**DOI:** 10.1155/2013/430413

**Published:** 2013-12-10

**Authors:** Matheus Diniz Gonçalves Coêlho, Francine Alves da Silva Coêlho, Ismael Maciel de Mancilha

**Affiliations:** ^1^FUNVIC, Faculdade de Pindamonhangaba, Laboratório de Parasitologia, Rodovia Presidente Eurico Gaspar Dutra s/n-Km 99, Pinhão do Una, 12422-970 Pindamonhangaba, SP, Brazil; ^2^Universidade de Taubaté, Campus do Bom Conselho, Laboratório de Parasitologia, Avenida Tiradentes 500, Centro, 12030-180 Taubaté, SP, Brazil; ^3^Universidade de São Paulo, Escola de Engenharia de Lorena, Laboratório de Probióticos Estrada Municipal do Campinho, s/n, 12602-810 Lorena, SP, Brazil

## Abstract

*Canis familiaris* is a domestic animal which plays an important role as a pet; however, it is very likely to develop diseases characterized by high morbidity and mortality. In this sense, there are some Ancylostomatidae species that can lead to anemia, weight loss, and abdominal pain. Therefore, the treatment with allopathic drugs is essential for eliminating the parasitism. However, the increasing occurrence of resistance to allopathic treatments stimulates the search for new therapeutic inputs, among which the probiotics stand out and are able to positively alter the intestinal microbiota and exert immunomodulatory effect. Therefore, the present study aimed to evaluate the applicability of using species of *Lactobacillus* (*L. acidophilus* ATCC 4536, *L. plantarum* ATCC 8014, and *L. delbrueckii* UFV H2B20) to control canine ancylostomiasis. A probiotic preparation containing 1 × 10^6^ CFU of each strain was administered to 10 naturally infected animals, on alternate days for 28 days. It was observed that the treatment with the probiotic preparation led to a significant reduction in EPG of feces (88.83%/ *P* < 0.05), as well as an increase in leukocyte and lymphocyte counts, highlighting the potential use of *Lactobacillus* species in the control of canine ancylostomiasis.

## 1. Introduction

Pets, especially domestic dogs, provide significant benefits to individuals and to society, since they can contribute to physical, social, and emotional development of children and the wellbeing of their owners; however, hygiene and dietary habits of the animals make them particularly prone to having a range of infectious diseases during their life [1, 2]. In this sense, especially the hookworms and the fact that they are identified as the most prevalent in these animals, as demonstrated in several parasitological surveys in Brazil and in the world [3–8]. The hookworms may cause intestinal disorders in dogs, leading to manifestation of symptoms such as anemia, weight loss, irritability, and abdominal pain and even result in death. They are also able to trigger pathological processes in humans such as cutaneous larva migrans and eosinophilic enteritis [9].

One of the most important steps to control hookworm is the adoption of antiparasitic treatment of infected dogs. However, despite the widespread availability of allopathic drugs for this purpose, there is a clear difficulty in decreasing the prevalence of this parasite in canine host [10]. Such issue can be attributed mainly to poor hygienic and alimentary habits of these animals and to the high environmental contamination. It is possible mainly by the existence of large amounts of stray dogs which are rarely treated and eventually spread helminth eggs and larvae [10]. In addition, treatment with antihelminthic commercially available requires the administration of several doses and a number of repetitions throughout the life of the animal, in case of being neglected or developing resistance [11, 12].

Therefore, it is necessary to develop new strategies to control this parasite, highlighting the potential use of probiotics for this purpose. Probiotics are products that consist of living and nonpathogenic microorganisms that can promote a balance in favor of the GI (gastrointestinal) tract microbiota, as well as an upregulation of the immune response, thereby contributing in the prevention and treatment of pathologies [13, 14]. In this regard, species of the genus *Lactobacillus* distinguish among microorganisms having probiotic properties, since they have the ability to tolerate acidic pH levels and, thus, to survive passage through the GI tract. These microorganisms also have the potential to inhibit or hinder the colonization of pathogenic species level in the digestive system, which can be evaluated as a potential tool for the control of various infectious processes afterwards, including the canine hookworm [14].

Thus, the present study aimed to evaluate the applicability of species of *Lactobacillus* having probiotic properties in the control of hookworm in dogs naturally infected.

## 2. Material and Methods

We evaluated the antiparasitic action of three strains of *Lactobacillus* administered as a “pool” to dogs infected with hookworm species from the Zoonosis Control Center (CCZ) of Taubaté, São Paulo, from January to March 2010. This paper was submitted to the ethics committee in animal research of FAPI and approved under protocol 009/2009.

Three strains of *Lactobacillus* were evaluated whose probiotic effects were demonstrated by Pereira [15] and Coutinho [16], namely: *Lactobacillus plantarum* ATCC 8014; *Lactobacillus acidophilus* ATCC 4536; and *Lactobacillus delbrueckii* UFV H2B20. *L. plantarum* and *L. acidophilus* strains were provided by the laboratory of Microbiology of the University Center Geraldo di Biasi, Volta Redonda, RJ, Brazil, and the *L*. *delbrueckii* UFV H2B20 strain was provided by the Department of Food Technology, from the Federal University of Viçosa, Viçosa, MG, Brazil.

All strains were maintained at 4°C on MRS agar (Man, Rogosa and Sharp) and were reactivated every 15 days in MRS broth, pH 6.2, sterilized at 121 for 15 minutes, and incubated at 37°C for 24 hours. The probiotic preparation was administered to animals in the form of “pool,” composed by mixing 1 mL of each culture containing 1 × 10^6^ colony forming units (CFU) of each strain. In order to check the absence of contamination of the strains, gram staining and catalase test were performed during the assays.

The amount of 1 × 10^6^ CFU was determined based on Technical Regulation of Bioactive Substances and Probiotics Isolates on Allegation of Functional and Health, according to which a preparation can only be considered as having probiotic property if it shows therapeutic property in a concentration of at least 1 × 10^6^ UFC/mL [17]. For experimental groups, animals with the following characteristics were selected: both genders, older than one-year old, without any diagnose or overt disease, and in whose feces only eggs of the Ancylostomatidae family species were found by coproparasitological examinations conducted in the laboratories of parasitology from Faculdade de Pindamonhangaba (FAPI) and from Universidade de Taubaté (UNITAU).

The study excluded pregnant female dogs, one-year old or less canids, and the ones in whose fecal samples structures compatible with any intestinal parasite were found, except the Ancylostomatidae family, as well as those with clinical symptoms or confirmed diagnosis of any infectious or severe disease. Parasitized animals that have recently undergone treatment with antiparasitic drugs or who have undergone this type of treatment in a period shorter than 60 days were also excluded from the experiment. Starting from the assumption that tests in Parasitology often require the use of nonparametric methods for evaluating treatments and, therefore, each experimental group must contain at least six observations [18], the number of dogs in each group was established as 10 in this study and each group was set clustered in separate kennel.

For the selection of the animals, fecal examinations were performed before the intervention using the Willis method [19]. The collection of fecal material was performed directly on the rectal ampulla of dogs and the samples deposited on stool specimen collectors, without added preservatives. Fecal material was sent under refrigeration to the laboratory of Parasitology of FAPI and UNITAU, using the method mentioned above. After coproparasitological evaluation, the number of eggs per gram of feces (EPG) of each animal selected for experimental groups was determined by the Gordon-Whitlock method [20].

After the selection of infected animals, they were grouped as follows: Group (A) 10 animals infected with Ancylostomatidae treated with the probiotic preparation under study; Group (B) 10 animals infected with Ancylostomatidae and not submitted to any treatment. The probiotic preparation under study, consisting of a “pool” of *Lactobacillus* strains previously grown in MRS broth, was diluted in skim milk and administered on alternate days to the animals for a period of 28 days. To assess the efficacy of treatment with the probiotic preparation, a count of parasitic structures in the feces of animals was performed 1 day before the experiment and 7, 14, 21, and 28 days after the start of the experiment, respectively, using the Gordon-Whitlock method [20] and, particularly for group (A), fecal samples were collected 14 days after completion of treatment to determine the EPG number. The percentage of EPG reduction was determined by using the EPG mean values of the first day before the treatment started (day zero) and the means of the respective samples, using the following formula, were described by Jacobs et al. [21]:

 % of EPG reduction = EPG mean (day 0) − EPG mean (day of interest) × 100/EPG mean (day 0).

In order to assess the evolution of the immune and anemic status, blood samples were collected from the animals in tubes containing EDTA (one day before submitting to treatment and 14 and 28 days after starting the experiment) and blood counts were determined. The erythrocyte count and total leukocyte count were performed in a Neubauer chamber [21]. The differential leukocyte count was performed on blood smears stained by Panoptic method [22]. The hematocrit or fused cell volume was determined, together with hemoglobin, by an automated method using a Coulter ACT8 equipment.

The results were evaluated according to the characteristics of the sample distribution. Parametric (ANOVA and Student's *t*-test) and nonparametric tests (Mann-Whitney and Kruskal-Wallis) were used, being applied according to the normality of the results, in a level of significance of 5%, using the software BIO ESTAT 5.0 as a support tool.

## 3. Results and Discussion

**Figure 1:**
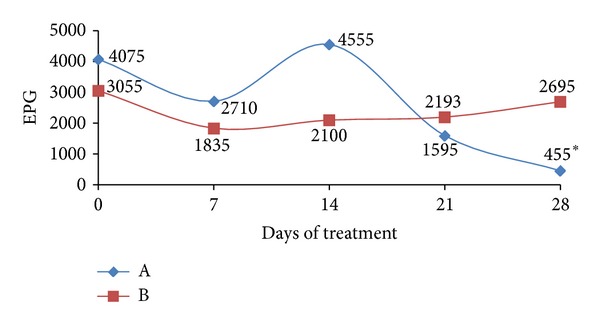
Mean number of eggs per gram of feces (EPG) in groups of dogs naturally infected with Ancylostomatidae: treated with probiotics (A) and untreated (B). *meaning difference in the reduction (*P* < 0.05).

One day before the start of the treatment (day 0), the EPG values from the control of infected untreated group (B) and treated with probiotics group (A) did not differ significantly (*P* > 0.05) from each other (Figure 1). However, on the 28th day after the treatment started, the mean of group (A) was 455 EPG, showing a reduction of 88.83% in fecal egg count (Table 1), which is statistically significant (*P* < 0.05) compared to that observed in the control group (B).

**Figure 2:**
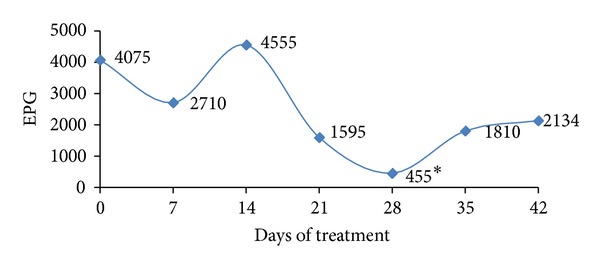
EPG mean values in the group of animals naturally infected and treated with probiotic preparation (A). *meaning difference in the reduction (*P* < 0.05).

The animals of group (A) were monitored regarding the egg laying in the feces, for 14 more days after the withdrawal of the treatment with the probiotic preparation. Therefore, the EPG determination was performed on days 35 and 42 and the results are shown in Figure 2. An increase in EPG values when compared to those determined on day 28, which suggests that it is of critical importance the inclusion of probiotic preparations in the diet, to ensure the maintenance of low counts of EPG.

As far as we know, this is the first report that shows the potential applicability of *Lactobacillus* species in the control of canine hookworm. The potential use of the strains evaluated in this study, regarding the antiparasitic activity, was also demonstrated by Countinho [16], which evaluated the control of cryptosporidiosis in experimentally infected mice. This author evaluated a probiotic preparation containing 1 × 10^12^ CFU of the same strains used in this study, which was administered for 10 consecutive days and observed 100% reduction of *Cryptosporidium parvum* oocysts in the feces of mice.

The positive effects of probiotics against parasitic infections have also been demonstrated in infections caused by species of nematodes of the family Ancylostomatidae. Mussi et al. [23] administered a combination of anthelmintic drugs (febantel, pyrantel pamoate, and praziquantel), along with a probiotic preparation of commercial use, consisting of *Lactobacillus acidophilus*, *Bifidobacillus bifidum*, and *Enterococcus faecium*, and although the effects of administering microorganisms have not been evaluated in isolation, these authors observed that the use of the probiotic product had a positive influence on the clinical conditions of dogs treated. Furthermore, Martínez-Gómez et al. [24] demonstrated the efficacy of administering probiotics to control another species of nematode, namely, *Trichinella spiralis* when administering 1 × 10^8^ CFU of *L. casei Shirota*, intraperitoneally, once a week for 3 weeks to 60 mice challenged with 200 larvae of *T. spiralis*. The authors noticed that there was a significant decrease in the number of adult parasites in the gut of animals.

**Figure 3:**
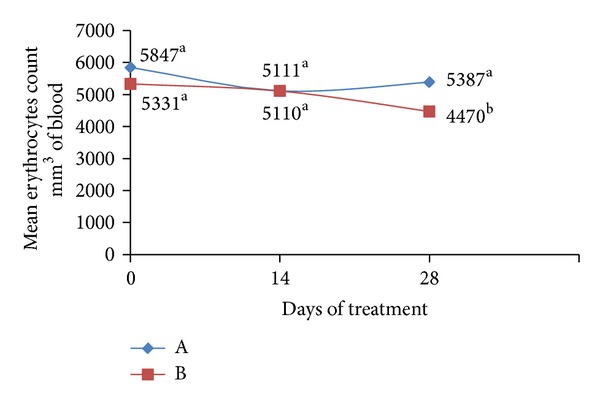
Mean values of erythrocytes count (×10^3^) in the peripheral blood of animals treated with probiotics (A) and nontreated animals (B). ^a,b^different letters represent significant differences in statistical terms (*P* < 0.05). Reference values: 6000–8000 × 10^3^/mm^3^ of blood.

In the present study, the ability of probiotic preparation in order to influence the anemia of infected animals was also evaluated. Before the treatment (T0), all dogs were anemic concerning the erythrocytes blood count observed when compared with reference values (6000 a 8000 × 10^3^ per mm^3^ of blood). There was no significant statistical difference regarding the number of red blood cells among the animals of group (A) and group (B) (Figure 3). However, after 28 days of treatment, animals in group (B) had significantly lower erythrocyte counts (*P* < 0.05) than observed in Group (A), indicating that the probiotic treatment was effective to stabilize the anemia that resulted from spoliation blood promoted by hookworms.

This stabilization of anemia may be associated with the 88.83% reduction of the number of EPG observed in group (A), possibly consequent from the declining number of parasites in the gut with consequent reduction of the spoliation of blood, and may also be related to the own action of the *Lactobacillus* species assessed. According to Silva et al. [25], microorganisms with probiotic property are able to increase the bioavailability of absorbable iron. The increase in the number of erythrocytes is linked with the increase of iron absorption which can be increased by the action of probiotics [25, 26]. Once absorbed, iron binds to transferrin transport iron to the bone marrow where erythroid precursors capture this metal for the production of the hemoglobin molecule. After this step, the erythroid precursors get mature and form red blood cells (erythrocytes), which migrate into the circulatory system. With this result, the greater the absorption of iron is, the greater the number of erythrocytes and the concentration of serum hemoglobin produced will be [27, 28].

**Figure 4:**
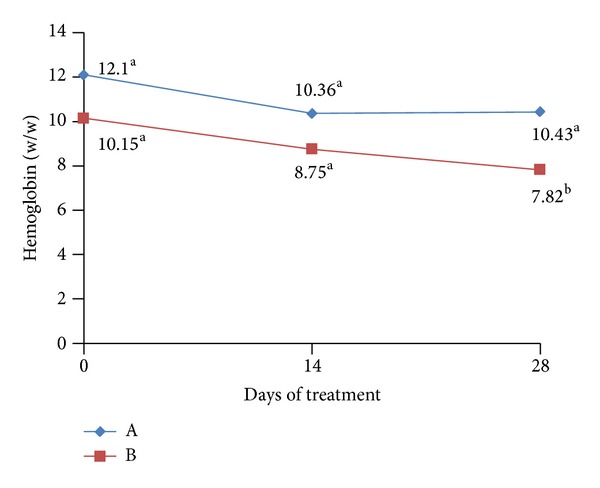
Mean rates of hemoglobin in the peripheral blood of animal groups assessed. ^a,b^different letters represent significant differences in statistical terms (*P* < 0.05). Reference values: 14–18 g (w/w).

The positive effects of administration of probiotic preparation may also be noticed when evaluating the concentration of hemoglobin in animals from both groups, as shown in Figure 4. It is observed that at the end of treatment (T28), there was a significant reduction in the hemoglobin concentration in group (B) probably related to spoiling of blood which is caused by parasites in the intestinal mucosa. Moreover, the reduction of the parasitic load induced by the administration of probiotics to group (A), probably induced a lower theft of blood, allowing a nonsignificant decrease in hemoglobin levels. There were no significant differences among the groups regarding the hematocrit levels.

**Figure 5:**
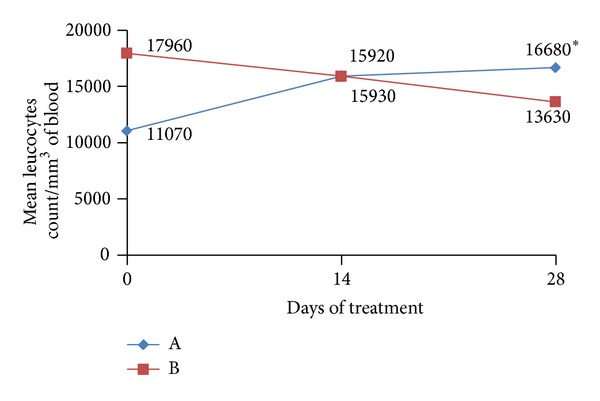
Mean values of leukocytes count in the peripheral blood of animal groups assessed. *significant difference within the group (*P* < 0.05). Reference values: 8000–16000 cells/mm^3^ of blood.

The amount of white blood cells as well as the differential count of leukocytes are two determinations that make the CBC (Complete Blood Count), and in this study, they were used as a tool to assist in the understanding of the mechanisms involved in the healing process of the canine hookworm, induced by probiotic preparation under study and the average results of the global score are shown in Figure 5.

After assessing the variations of global values of leukocytes in group (A), it is possible to show that the leukocyte count observed during the last determination (day 28) was significantly higher (*P* < 0.05) than the one observed on the first day (day 0) and second week (day 14) of experimental monitoring. This fact may have influenced positively in the reduction of OPG that was observed in this group. However, it should be emphasized that although there was an upward tendency in the number of leukocytes only in the treated group, there was no statistical difference between the two groups. Concerning the reference values, there was a contrasting situation: in the beginning of the treatment (day 0), the meaning value of the leucocyte of the group not treated was above the normal value and in the end of treatment (day 28) this occurred only in the treated group. According to Gill [29], the consumption of fermented milk containing probiotic microorganisms, particularly *Lactobacillus* species, may improve the function of the innate immune response, increasing the number of leukocytes and phagocytic activity of peripheral blood leukocytes which can remain elevated for several weeks, even after consumption ceased. That can positively influence the immune response for protection against infections as well.

**Figure 6:**
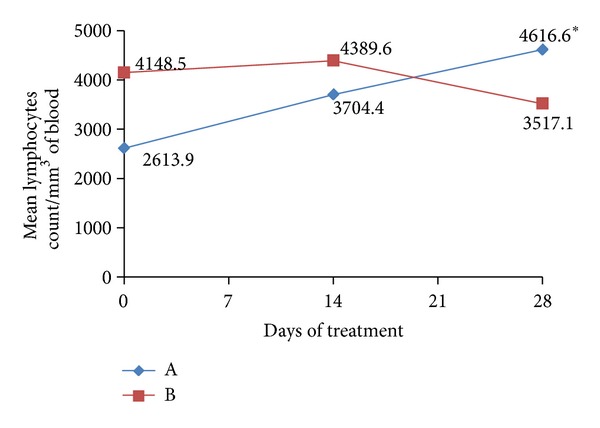
Mean values of lymphocytes count in the peripheral blood of animal groups assessed. *significant difference within the group (*P* < 0.05). Reference values: 800–4160 cells/mm^3^ of blood.

It is also verified (Figure 6) that the average values of lymphocytes observed in group (A) after 28 days were significantly higher than those observed on days 0 and 14 of treatment, as well as higher than the reference values (800 to 4160 lymphocytes per mm^3^ of blood), characterizing lymphocytosis. This significant increase in the number of lymphocytes was observed only in the animals of this group, thus emphasizing that the administration of probiotics positively influenced the immune response by stimulating the production of lymphocytes. This hypothesis is strengthened by the fact that the peak increase in the number of lymphocytes in group (A) coincideds with the period in which a significant decrease in the number of EPG in the feces (Figure 6) was evidenced, which occurred on the day 28 of experiment.

With respect to other subtypes of leukocytes, among which neutrophils and eosinophils, there were no statistically significant changes in their scores during the experiment.

## 4. Conclusion

The administration of the probiotic preparation containing 1 × 10^6^ UFC of *Lactobacillus plantarum* ATCC 8014; *Lactobacillus acidophilus* ATCC 4536 and *Lactobacillus delbrueckii* UFV H2B20 to the infected dogs induced a significant increase in global leukocyte and lymphocyte counts and a decrease in the number of EPG in feces after 28 days of treatment. However, after this period, the EPG values tended to increase, thereby emphasizing the importance of the inclusion of a probiotic preparation in animal diets as an alternative to keep them free of parasites.

## Figures and Tables

**Table 1:** EPG reduction (%) in groups of dogs naturally infected with Ancylostomidae: treated with probiotics (A) and untreated (B).

	EPG reduction (%)				
	day 0	day 7	day 14	day 21	day 28
A	0	33,49%	−11,77%	60,85%	88,83%*
B	0	39,93%	31,26%	28,01%	11,78%
